# Changes of Gut-Microbiota-Liver Axis in Hepatitis C Virus Infection

**DOI:** 10.3390/biology10010055

**Published:** 2021-01-13

**Authors:** Mohammed El-Mowafy, Abdelaziz Elgaml, Mohamed El-Mesery, Salma Sultan, Tamer A. E. Ahmed, Ahmed I. Gomaa, Mahmoud Aly, Walid Mottawea

**Affiliations:** 1 Department of Microbiology and Immunology, Faculty of Pharmacy, Mansoura University, Mansoura 35516, Egypt; seven@mans.edu.eg (M.E.-M.); elgamel3a@mans.edu.eg (A.E.); 2 Department of Microbiology and Immunology, Faculty of Pharmacy, Horus University, New Damietta 34518, Egypt; 3 Department of Biochemistry, Faculty of Pharmacy, Mansoura University, Mansoura 35516, Egypt; m_elmesery@mans.edu.eg; 4 School of Nutrition Sciences, Faculty of Health Sciences, University of Ottawa, Ottawa, ON K1H 8M5, Canada; ssult017@uottawa.ca (S.S.); tahmed@uottawa.ca (T.A.E.A.); agomaa@uottawa.ca (A.I.G.); maly@uottawa.ca (M.A.); 5 Department of Cellular and Molecular Medicine, Faculty of Medicine, University of Ottawa, Ottawa, ON K1H 8M5, Canada; 6 Department of Nutrition and Food Science, National Research Center, Doki, Cairo 12622, Egypt; 7 Department of Biology, School of Health Science and C.J., The State University of New York Canton, Canton, NY 13617, USA

**Keywords:** gut microbiota, gut liver axis, HCV, dysbiosis, antiviral drugs

## Abstract

**Simple Summary:**

Gut microbiota alteration is linked to many health disorders including hepatitis C virus (HCV) infection. This dysbiosis in turn impacts the coordination between the gut and the liver that is known as the gut–liver-axis. Here, we discuss the latest findings regarding the changes in gut microbiota structure and functionality post HCV infection and its treatment regimens. In addition, we underline the contribution of the microbiota alterations to HCV associated liver complications.

**Abstract:**

The gut–liver-axis is a bidirectional coordination between the gut, including microbial residents, the gut microbiota, from one side and the liver on the other side. Any disturbance in this crosstalk may lead to a disease status that impacts the functionality of both the gut and the liver. A major cause of liver disorders is hepatitis C virus (HCV) infection that has been illustrated to be associated with gut microbiota dysbiosis at different stages of the disease progression. This dysbiosis may start a cycle of inflammation and metabolic disturbance that impacts the gut and liver health and contributes to the disease progression. This review discusses the latest literature addressing this interplay between the gut microbiota and the liver in HCV infection from both directions. Additionally, we highlight the contribution of gut microbiota to the metabolism of antivirals used in HCV treatment regimens and the impact of these medications on the microbiota composition. This review sheds light on the potential of the gut microbiota manipulation as an alternative therapeutic approach to control the liver complications post HCV infection.

## 1. Clinical Manifestations of HCV Infection

### 1.1. Hepatitis C Virus Structure

Advances in molecular biology techniques have led to detecting the first incidence of Hepatitis C Virus (HCV) infection in 1989 [1]. HCV is a single-stranded RNA virus that belongs to the Flaviviridae family and genus Hepacivirus [2]. HCV genome size is 9600 nucleotides, that encode about 3000 amino acids [3,4]. Viral replication and translation rely on the 5′- and 3′- untranslated region (UTR) as they are the initiation sites of viral RNA replication [5,6]. The open reading frame of HCV is characterized by encoding ten different proteins, including structural and nonstructural proteins produced from proteolytic cleavage of a polyprotein starting at 5′-UTR internal ribosomal entry site. The structural proteins include core, and the envelope glycoproteins E1 and E2 proteins, while the nonstructural proteins (NS) are p7, NS2, NS3, NS4A, NS4B, NS5A, and NS5B [3]. Another recently detected protein is produced due to a shift in the coding region of the core protein, the F protein [7]. The core protein has several functions, including constructing the viral capsid and interaction with other cellular proteins and signaling pathways involved in the HCV life cycle [8]. The two envelope proteins, E1 and E2, are essential for viral envelope formation, viral binding, and viral entry into host cells [9,10]. E2 is the key player in viral attachment through interaction with the cellular receptor complex. E1 and E2 are also essential for ER localization and heterodimer assembly. P7 is a small transmembrane protein that is essential for the virus infectivity. A mutation in its cytoplasmic loop suppressed the transfection of HCV cDNA in chimpanzees. NS2 is responsible for combination with the endoplasmic reticulum (ER), where it acts as zinc-dependent metalloprotease together with the N terminal domain of NS3 [11]. NS3 has a serine protease domain in the N terminal and helicase/NTPase domain in the C terminal [7]. NS4A is characterized by a central part of 21–30 amino acids that acts as a cofactor for the NS3 protease activity [12]. NS3-NS4A protease catalyzes the cleavage of the viral polyprotein into NS3/NS4A, NS4A/NS4B, NS4B/NS5A, and NS5A/NS5B proteins. NS4B is a protein composed of 261 amino acids and has several functions, such as participation in replication complex and controlling the activity of RNA-dependent RNA polymerase (RdRp) of NS5B [13]. NS5A proteins contribute to viral replication and interact with cellular proteins such as IFN-α protein kinase, causing resistance to IFN-α as an anti-viral drug [14]. NS5B is an RdRp that is a tail-anchored protein and acquires a right-hand structure that can be divided into palm, finger, and thumb domains [15,16]. The p7 protein is composed of 63 amino acids and is essential for HCV to infect hepatocytes [17]. This is a brief description of HCV proteins, while the functions and characteristics of these proteins have been extensively reviewed before [18].

### 1.2. Infection with HCV

Infection with HCV occurs primarily due to the contact with infected blood that may occur during injection or blood transfusion [19,20]. Developing countries with low resources such as countries of the Middle East, North Africa, India, and East Asia represent more than 80% of the viral burden worldwide, with prevalence rates of 3.6% in North Africa/Middle East and 3.7–3.8% in central and East Asia [21,22]. Although tests for HCV detection reduce infection incidence in these countries, poor medical procedures are considered the leading cause of newly infected patients [19,20]. Interestingly, the complete life cycle of HCV occurs only in the hepatocytes and not in other cell types of the human liver [23,24]. Accordingly, liver complications in HCV infections are due to the developing of inflammation in hepatocytes that may progress into liver fibrosis. Further liver damage may lead to the development of cirrhosis and hepatocellular carcinoma (HCC), which are considered the leading cause of death at the late stage of the infection [20,25]. Indeed, the incidence of cirrhosis development in HCV-infected patients is up to 18% after 20 years of the infection and may reach more than 40% after 30 years of the infection as previously reported by a broad meta-analysis study [26].

### 1.3. HCV Genotypes and Epidemiology

Phylogenetic analysis of HCV genotypes has confirmed the existence of 7 main genotypes and 67 subtypes [27]. The most predominant HCV genotypes are the genotype 1 and the genotype 3, accounting for about 46% and 30% of the infection incidence, respectively [28,29]. Genotype 1 is most predominant in Europe followed by genotypes 2 and 3. The most infection cases of genotype 2 are in central Africa, while genotype 3 represents the predominant genotype in several countries such as India, Bangladesh, Thailand, and Pakistan. The highest incidence of HCV infections with genotype 4 were recorded in Middle East and central Africa, while southern Africa is the region that exhibits a high incidence of genotype 5. East and Southeast Asia, such as Laos and Vietnam, are reported to have the highest incidence of genotype 6 [29]. Genotype 7 is the one with the rare incidence and was detected only in a few cases in central Africa [30]. Indeed, the development of different HCV genotypes can be attributed to high error incidence of RNA-dependent RNA polymerase and the pressure induced by the host immune defense [31]. The seven HCV genotypes differ in about 30–35% of the total number of nucleotides, while the sub-genotypes differ in 20–25%, particularly in the regions encoding glycoproteins such as E1 and E2 [4,32].

Historically, HCV epidemiological infection with genotype 3a started to increase in India in 1940 [33]. Infection with HCV genotype 4 started in central Africa accompanied by the transport of several lineages to other lands in North Africa such as Egypt, which is reported for the predominance of genotype 4a [34]. Vietnam was reported for the increase in HCV infection with several subtypes of genotype 6 (6a, 6e, 6h, 6k, 6l, 6o, and 6p) and also with sub-genotypes 1a and 1b. The incidence of sub-genotypes 1a and 1b in Vietnam started to increase from 1955 for several years until 1984, during the period of the Vietnam War [35].

Although the main relevance of the HCV genotypes is mainly illustrated as an epidemiological marker, infections with different HCV genotypes may exhibit variable clinical features. For example, it has been reported that genotype 1 is characterized by a high HCV RNA in the serum of infected patients [36]. Additionally, patients infected with genotype 2a showed higher response rate to IFN-α treatment than those infected with genotype 1b [37]. It was proven that the incidence of nonalcoholic fatty liver disease increases in patients infected with genotype 3 [38]. Genotypes 2 and 4 are associated with the incidence of insulin resistance [39]. Thus, the relevance of genotype identification can be summarized in 4 points: (1) As an epidemiological marker; (2) variability of responses to the available treatment strategies; (3) different genotypes induce variable degree of pathogenesis; (4) variable sensitivity and specificity of the diagnostic methods to different genotypes [40].

### 1.4. Chronic HCV Infection and Stages of Liver Damage

In 2019, about 71 million people were infected with HCV globally, and among them, 400,000 infected people died due to liver damage associated with HCV infection [41]. Chronic HCV infection induces inflammation in hepatocytes that usually leads to fibrosis induction. Although inflammation is considered a normal defense mechanism against the hepatocytes’ viral infection, continual unregulated inflammation is the main criteria of the chronic liver damage induced by HCV infection, including fibrosis, cirrhosis, and HCC [42,43]. Chronic HCV infection is characterized by the release of different inflammatory mediators expressed by hepatic cells, kupffer cells, and immune cells employed by the liver, leading to induction of inflammatory responses [44,45]. One known inflammatory mediator that play a crucial role in fibrosis induction is TGF-β1, which induces fibrogenesis-related gene expression of hepatic stellate cells (HSCs) [46]. Another inflammatory mediator is the NOD-like receptor family pyrin domain containing 3 (NLRP3), which participates in HSCs activation and fibrosis induction [47].

The prolonged induction of inflammatory signals in hepatocytes leads to the stimulation of HSCs to secrete extracellular matrix (ECM) in the liver that results in the induction of liver fibrosis and cirrhosis. Indeed, a scar formation may arise due to increased ECM deposition in the liver that can be recovered by the process of fibrolysis at the early stage of HCV infection [41]. However, chronic accumulation of the liver damage and ECM accumulation in the liver leads to an imbalance between fibrogenesis and fibrolysis with an increased rate of fibrogenesis that may lead to fibrosis progression into cirrhosis with irreversible liver damage [41]. Liver cirrhosis is an advanced stage of fibrosis that also includes a distortion of hepatic vasculature, mainly by linking the portal tract with each other and with the central vein [48].

One of the known complications in cirrhotic HCV-infected patients is the changes in portal microcirculation resulting in thrombin formation and microthrombosis of portal vein radicles [49]. As mentioned before, HCV proteins influence signaling pathways involved in several vital processes that may interfere with other pathological conditions such as insulin resistance. Therefore, patients who are chronically infected with HCV are at risk of developing insulin resistance and impaired glucose metabolism that may be worsening at advanced stages of liver fibrosis [50,51].

### 1.5. Mechanism of HCC Development in HCV Infected Patients

Although cirrhotic liver in chronic HCV infected patients may progress to HCC development, HCV is classified as an indirect oncogenic virus based on the very low percentage of HCC development in HCV-infected patients and its inability to infect HCC cancer cells rather than normal hepatocytes [52]. Moreover, HCV is an RNA virus that is unable to be incorporated in the host genome, unlike the hepatitis B virus (HBV), and therefore, HCV is considered as an indirect oncogenic virus [53].

HCV replication in hepatocytes leads to the accumulation of several viral proteins and positive-strand RNA in ER that induces remodeling of the ER membrane to acquire proteins required for viral replication and assembly [54]. Such impact mediates the signaling pathways of cell injury and inflammation during fibrosis, cirrhosis, and HCC [55]. Thus, induction of ER stress is considered as one of the proposed mechanisms for the ability of HCV to induce HCC. Another explanation of HCC development in HCV-infected patients is the ability of HCV proteins such as NS5B polymerase to bind to the retinoblastoma tumor suppressor protein, which controls hepatic cell proliferation [56,57]. Additionally, other HCV proteins such as NS2 and core proteins may influence cell cycle progression. Aggressive HCC development has also been reported due to interference of the RAF/MAPK/ERK pathway by many HCV proteins, including core, E2, NS3, and NS5A that trigger cellular proliferation [58].

## 2. Gut Microbiota Structure and Contributions to Human Health

### 2.1. Definition of Microbiome and Microbiota

Microorganisms do not live alone, rather they live in communities. Moreover, they are closely associated with advanced living beings, including humans, animals, and plants. Within these organisms, microorganisms live in aggregates and establish different relationships involving mutualism, commensalism, amensalism, and/or parasitism [59]. The collection of microorganisms sharing the same environment is typically named microbiota, while microbiome refers to the collective genomes of the microbes [60]. In the human body, the microbiota exists everywhere from the skin to the gut, and even in regions thought to be sterile including blood [61]. There are excessive data stating that more than 10,000 microbial species reside in different areas of the human body [62,63]. The diversity of these microbes differs from one site to another, recording the lowest diversity in organs like the skin and the highest diversity in other organs, particularly the gut [64]. It is well documented that there is an important effect of the microbiota on human health and disease states [65,66,67,68].

### 2.2. Gut Microbiota

Anatomically the gut is defined as the tract from the mouth to the anus. The gut’s primary function is the digestion of food and absorption of nutrients [69]. In the mouth and pharynx region, the microbiota is composed mainly of commensal bacteria belonging to the genera *Moraxella*, *Haemophilus*, *Neisseria*, and *Streptococcus*. In the small intestine and stomach, few species of bacteria are found, while most microbes reside in the colon (around 10^12^ cells/g) [69]. It is noteworthy that there are many microorganisms symbiotically occupying the gut together with the bacteria including archaea, fungi, viruses, and protists [69]. Regarding bacteria, the predominant four phyla found in the gut are Bacteroidetes, Firmicutes, Actinobacteria, and Proteobacteria [70]. The major remarkable bacteria belong to the genera *Bacteroides*, *Bifidobacterium*, *Clostridium*, *Ruminococcus*, *Peptostreptococcus*, *Faecalibacterium*, *Peptococcus*, and *Eubacterium* [71]. Along with bacteria, the most common coexisting fungal genera in the gut are *Saccharomyces*, *Candida*, *Galactomyces*, *Pleospora*, *Bullera*, *Aspergillus*, *Trametes*, *Sclerotinia*, *Penicillium*, and *Rhodotorula* [72].

### 2.3. Role of Gut Microbiota in Host Immune System and Metabolism

Gut microbiota plays many beneficial roles in human health. First, they are essential for the shaping and evolution of the immune system [73]. Germ-free mice often exhibit defective immune organs and cells including abnormal gut-associated lymphoid tissues (GALT), smaller and fewer Peyer’s patches, less cellular lamina propria, fewer cellular mesenteric lymph nodes, lower expression of Toll-like receptors (TLRs) and major histocompatibility complex class II (MHC II) molecules, invariant natural killer T cells (iNKT), and reduced intraepithelial lymphocytes and CD4+ T cells compared to microbiota-colonized mice [73,74]; gut colonization of these germ-free mice with a conventional microbiota mitigates these deficiencies [73,74]. Additionally, a study showed that mice receiving CD4 + CD62L + lymphocytes from germ-free mice developed colitis faster than mice receiving the same TReg cells from conventionally housed mice [75]. Together, these data suggest that gut microbiota plays critical roles in the development of the intestinal immune system.

In addition to immune system maturation, some microbiota components, such as *Bacteroides*, *Fusobacterium*, *Propionibacterium*, and *Eubacterium*, are essential for the synthesis of vitamins such as vitamin B and K [76]. Gut microbiota is also involved in the metabolism of non-digestible carbohydrates to yield short-chain fatty acids (SCFAs), which are important to maintain colon health and metabolic homeostasis [77]. Microbiota-generated SCFAs mainly include butyrate, propionate, and acetate. Butyrate is primarily utilized by colonocytes as a main energy source, while acetate and propionate act as substrates of lipogenesis and gluconeogenesis in peripheral tissues [78,79]. SCFAs also contribute to the integrity of the intestinal barrier. For instance, butyrate upregulates the expression of tight junction-associated proteins [80]. In addition to colonic fermentation of dietary fibers, gut microbiota interacts with other host metabolic processes such as regulating bile acid metabolism, metabolism of choline, and insulin resistance [81]. It is worth mentioning that many previous reports confirmed that normal performance of the hepatic system is related to nutrients obtained from the metabolic actions of the gut microbiota [74,82,83,84,85,86].

## 3. Gut–Liver Axis

The gut–liver axis represents the link between the gut microbiota and an extraintestinal organ, the liver, where both communicate via the portal vein, systemic circulation, and biliary tract [81]. Portal circulation through the portal vein is responsible for most anatomical interactions and communications between the liver and the gut. It supplies about 70% of the blood to the liver and, therefore, is responsible for transporting nutrients and metabolites produced in the gut to the liver [82]. The gut microbiota and host cells metabolize nutritional macromolecules such as carbohydrates, lipids, and proteins. The metabolic products may be transferred to the liver through the portal vein [83]. This communication also leads to the transport of toxic byproducts of the gut microbiota to the liver such as peptidoglycans, endotoxins, or the intact bacteria, which may disrupt metabolic functions of the liver [87]. On the other side, bile acids are generated in the liver and then released into the intestine, and therefore they get involved in the regulation and communication of the gut–liver axis [84,88]. Portal circulation through the portal vein is responsible for most anatomical interactions and communications between the liver and the gut. It supplies about 70% of the blood to the liver and, therefore, is responsible for transporting nutrients and metabolites produced in the gut to the liver [89]. The gut microbiota and host cells metabolize nutritional macromolecules such as carbohydrates, lipids, and proteins. The metabolic products may be transferred to the liver through the portal vein [90]. As mentioned above, this communication also leads to the transport of harmful byproducts of the gut microbiota to the liver, which may disrupt metabolic functions of the liver [91]. On the other side, bile acids are generated in the liver and then released into the intestine, and therefore they get involved in the regulation and communication of the gut–liver axis [92].

### 3.1. Bile Acids and Gut-Liver Axis

Synthesis of bile acids occurs in the liver from cholesterol to produce the primary bile acids, including cholic acid and chenodeoxycholic acid. These bile acids are transported to the duodenum by the biliary tract where they are converted by 7α-dehydroxylation and deconjugation into secondary bile acids including deoxycholic acid and lithocholic acid. Such bile acid conversion is mediated by some gut microbes, mainly Clostridiales [93,94]. Bile acids have crucial roles in food digestion, keeping the integrity of gut mucosa and having antimicrobial activity against invading pathogens [94,95]. It is well reported that late complicated cases of HCV infections, especially cirrhosis, are characterized by alteration of the conversion of primary bile acids into secondary bile acids; however, the mechanism of this alteration is still unknown [96]. One scenario that may explain the bile acid reduction with the progression of HCV infection may be because of gut microbiota dysbiosis, which eventually leads to decreasing the microbial diversity and increasing the abundance certain microbial taxa such as the Proteobacteria phylum, *Enterobacteriaceae family*, and the genera *Staphylococcus* and *Enterococcus* [96,97].

Bile acids have attracted considerable research interest in HCV treatment due the reported defect in the treatment with IFN therapies in patients who have high serum levels of bile acids [98,99]. On the other side, HCV treatment with direct-acting antiviral (DAA) was associated with a four-fold increase in the total bile acid levels by week 4 antiviral therapy [100]. Moreover, ritonavir was reported to induce changes in bile acid transport [100]. Regarding the functions of bile acids, it is well known that bile salts play a vital role in absorption and digestion of lipid components in addition to their role as ligands for nuclear receptor known as farnesoid X receptor (FXR) that controls several genes required for the metabolism of different macromolecules such as lipids, glucose, and bile acids [101]. It was reported that bile salts lead to activation of FXR that was associated with the activation of lipoprotein lipase, which induces entry of HCV to different cell types and interferes with its infection [102]. Another study reported that bile acids induce HCV replication depending on EGFR/ERK pathway that affects the efficacy of anti-viral treatment drugs [103]. There are previous studies that reported the role of bile acids in the replication of HCV genotype 1 [104,105]. Another study detected the ability of bile acids to increase genotype 1 and genotype 2a replication in Huh7 and Huh6 cell lines, but with lower capacity in the case of genotype 2a that suggest the interference of bile salts with anti-viral treatment and their role to improve HCV replication in vitro in cell lines [106].

### 3.2. Intestinal Barrier

The intestinal barrier prevents toxic compounds such as bacteria and their byproducts from reaching extraintestinal body organs and other tissues including the liver. Thus, impaired intestinal barrier exposes the liver to harmful and toxic compounds from the intestine, which may cause liver damage and impairment of its function, such as alcoholic liver disease, primary biliary cholangitis, and liver cirrhosis [107]. For instance, the increase in intestinal permeability, associated with gut microbiota dysbiosis, exposes hepatocytes pathogen-associated molecular patterns (PAMP), and damage-associated molecular patterns that contribute to liver injury [108]. PAMPs have a direct effect either on hepatocytes or innate immune cells in the liver, such as kupffer and stellate cells [109]. Accordingly, the above discussed findings illustrate that the relationship between the liver and the gut microbiota is bidirectional, and gut microbiota dysbiosis is associated with liver disorders. However, the mechanistic understanding of the cause–effect relationship requires further studies on a case-by-case fashion.

## 4. Gut Microbiota Dysbiosis in HCV

### 4.1. Gut Microbiota During Liver Disease Manifestations

Gut microbiota have been linked to fatty liver disease, autoimmune hepatitis, alcoholic liver disease, viral hepatitis (HBV and HCV), primary biliary cholangitis, and primary sclerosing cholangitis (PSC) [110,111,112,113,114,115,116]. Some gut microbes such as *Lactobacillus plantarum*, *Saccharomyces boulardii*, and *Bifidobacterium* species may mitigate different types of hepatitis and metabolic disorders [114]. The gut mucous membranes act as the entrance doors for invading pathogens [117]. In the case of viruses causing hepatitis, the virus shatters the intestinal mucosa and breaches the permeability, resulting in gut dysbiosis and proclamation of liver cirrhosis and HCC through inducing the release of pro-inflammatory cytokines [69]. Therefore, it is not surprising that mitigation of gut dysbiosis, for example, by using probiotics or prebiotics, helps lessen the tolerogenic response and improve mucosal protection against viral pathogens [118]. For instance, the increased abundance of *Lactobacillus* in the gut microbiota positively correlates with murine norovirus inhibition by vitamin A, through the upregulation of interferon (IFN-β) [119]. Moreover, a mixture of *Bifidobacterium* and various probiotics with fructo-oligosaccharides and galacto-oligosaccharides has a protective effect against Rotavirus infection in a rat model [118]. This is mediated by upregulating the expression of IFN-γ, IL-4, TNF-α, and TLR2 promoting their production [118]. The major complications of liver diseases, notably cirrhosis, are characterized by gut dysbiosis depicting an increase in the families of *Enterobacteriaceae*, *Veillonellaceae*, and Proteobacteria phylum, as well as a decrease in *Lachnospiraceae* family and the phylum of Bacteroidetes [120]. Consistently, the cirrhosis dysbiosis ratio is derived to describe the variations in gut microbiota in patients suffering from cirrhosis with beneficial *Ruminococcaceae* and *Lachnospiraceae* and harmful *Enterobacteriaceae* bacteria [121]. Other liver manifestations involving severe cirrhosis and hepatic encephalopathy are characterized by an elevation in *Enterobacteriaceae* levels [122].

### 4.2. Dysbiosis of Gut Microbiota During Hepatic Viral Manifestations

Hepatitis occurs due to infection with different viruses, including hepatitis A (HAV), HBV, HCV, or hepatitis E virus (HEV). Hepatitis represents an important health concern, particularly in developing countries [69]. HAV and HEV lead to acute manifestations that may be self-cured and short-lived except in immunosuppressed individuals. Taxonomically, HAV and HEV belong to RNA viruses that can be transmitted via ingestion of contaminated food and water and may severely impact intestinal microbiota [123]. A previous study demonstrated that HEV’s manifestations in pigs can be treated using probiotics containing *Enterococcus faecium* NCIMB 10415 [124]. Nonetheless, there is a deficiency of related records in humans. Besides, HAV and HEV, HBV, and HCV are serious health problems globally that are responsible for numerous reported cases of chronic hepatitis and death [125,126]. The major concern regarding these two viruses is their ability to establish chronic infections (at 10% in HBV and more than 30% in HCV) that finally result in severe complications, including cirrhosis and HCC [125,126]. In order to evade the immune system, hepatic viruses have developed many mechanisms, including dysbiosis of intestinal microbiota [127]. It is well documented that chronic hepatitis infections lead to massive translocation of the intestinal microbiota [128,129]. Such translocation impairs the primary barrier causing the growth of pathogenic bacteria at high rates, and abnormal regulation of immune cells that finally results in severe intestinal inflammation [130]. Moreover, the situation may worsen due to loss of homeostasis of the intestinal microbiota, which results in the progression of hepatitis viral infection [131]. Therefore, during chronic hepatic viral infections, microbiota have a great influence on viral replication as well as the interactions between host cells and the virus. During viral hepatitis, some taxa prevail including the family of *Enterobacteriaceae* and the species including *Enterococcus faecalis*, *Escherichia coli*, and *Faecalibacterium prausnitzii*, while others are reduced, such as lactic acid bacteria involving the genera of *Leuconostoc*, *Lactobacillus*, *Weissella*, and *Pediococcus* [121,132]. Moreover, prevalence of other bacterial species are related to liver complications (cirrhosis and/or HCC) following HBV or HCV infections, including bacterial families such as *Enterobacteriaceae*, genera including *Neisseria* and *Gemella*, and species like *E. faecalis*, *E. coli*, and *F. prausnitzii* [114,132]. Finally, a fungal pathogen, namely *Candida*, is reported in patients suffering from HBV related cirrhosis [133].

### 4.3. Dysbiosis of Gut Microbiota During HCV Infection

HCV is considered one of the major etiological agents of hepatitis, which results in severe liver complications, including cirrhosis, HCC, and it may even lead to liver failure and death [125]. There is little published literature regarding the impact of HCV infection on gut microbiota [97,121,134,135,136,137,138,139]. Additionally, the mechanistic understanding of how microbial dysbiosis participates in the disease’s progression is not completely addressed. It is well documented that dysbiosis can be classified into three main categories; (i) alteration of beneficial microorganisms, (ii) predominance of harmful microorganisms or pathobionts, and (iii) alteration of total microbial diversity [140]. The studies performed to reveal the effect of HCV on microbiota dysbiosis depicted lower bacterial diversity in HCV infected patients than healthy individuals. This diversity alteration is directly related to the severity and stage of the disease [112]. The previous finding may be attributed to immunoglobulin A (IgA) production by gastric B-lymphocytes infected by HCV that induces changes in the constitution of gut microbiota [141]. Interestingly, chronic liver diseases in advanced stages are associated with more alterations in gut microbiota than patients with the less developed disease [142]. This HCV-associated depletion of the gut microbiota diversity was mitigated after treatment with antiviral drugs [97]. In contrast, a recent study reported a lower microbial diversity in healthy adults as compared to treatment of naïve newly diagnosed HCV patients, which illustrates the impact of the treatment as a confounding factor in microbiome studies [137]. Additionally, this difference in HCV-associated diversity may be attributed to different cohort’s ethnicities, therapeutic factors, and different disease stages.

It was observed that during HCV infection, the order Clostridiales is significantly depleted, while both *Lactobacillus* and *Streptococcus* genera are significantly increased [69]. In addition, it is well documented that the phylum of Bacterioidetes, the family of *Enterobacteriaceae*, and viridans streptococci are increased in the case of chronic HCV patients, while the phylum of Firmicutes is decreased [112]. It is also noteworthy that HCV is associated with an increase in the lipopolysaccharides (LPS) serum levels, which indicates a damaged intestinal barrier and microbial translocation and inflammation during the disease progression [112,143].

The stages of HCV infection can be classified into (i) persistently normal serum alanine aminotransferase stage (PNALT), (ii) chronic hepatitis, (iii) liver cirrhosis, and (iv) HCC [112]. It is reported that there is a significant reduction in *Ruminococcaceae* and *Lachnospiraceae* families in chronic hepatitis, liver cirrhosis, and HCC patients. On the other hand, there is a significant increase in Lactobacilli and viridans streptococci. PNALT patients depict a significant increase in the genus of *Bacteroides* and the family of *Enterobacteriaceae*. Remarkably, *Streptococcus salivarius* is significantly increased in HCC patients with liver cirrhosis, indicating that *S. salivarius* may play a role in developing liver cirrhosis and progression to HCC [112]. Metabolic dysfunction of bile acids associated with HCV may be the key factor in the gut microbiota dysbiosis. This bile disturbance may result in overgrowth of proinflammatory bacteria, involving *Porphyromonadaceae* and *Enterobacteriaceae* families along with reducing the phylum of Firmicutes, the key producers of secondary bile acid generation [144]. In addition, *Ruminococcaceae* and *Lachnospiraceae*, the two Firmicutes families, are major SCFAs producers through fermentation of carbohydrates in human intestines [145,146]. Such SCFAs are crucial for the induction and differentiation of colonic regulatory T (Treg) cells responsible for suppressing inflammation [147,148]. SCFAs are also crucial for the nutrition of colon epithelia and adjusting its pH [149]. Thus, deprivation of SCFAs as a result of gut dysbiosis due to alteration of *Ruminococcaceae* and *Lachnospiraceae* would result in worsening the state of chronic HCV patients. This is mainly due to the loss of pH control, leading to hyperammonemia and ammonia absorption in the gut [150].

During both PNALT and liver cirrhosis, there is a significant increase in the genus of *Bacteroides* and *Enterobacteriaceae* family [97,123,151]. These reports explain that gut dysbiosis results in decreasing bile acids that finally alter the gut microbiota diversity [97,152]. In addition, the increase in *Bacteroides* and *Enterobacteriaceae* may explain the inflammation that occurs in patients suffering from hepatic encephalopathy [147,153]. Therefore, the increase in levels of *Enterobacteriaceae* as well as *Bacteroides,* may be used as a biomarker for proinflammation that finally may result in endotoxemia.

**Figure 1:**
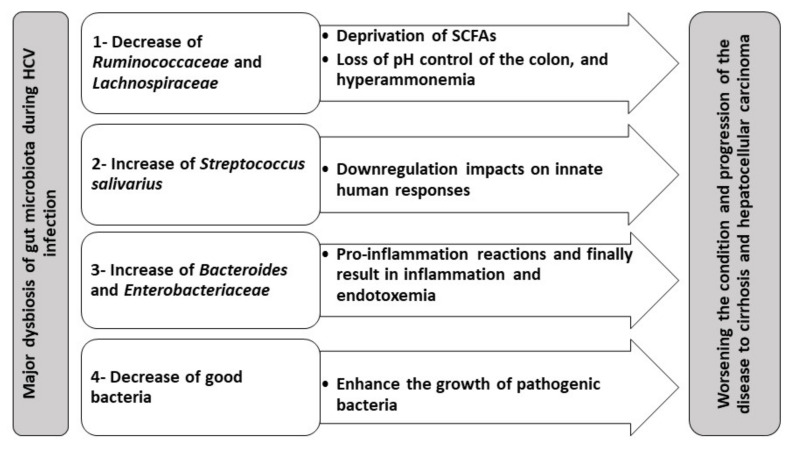
Gut microbiota dysbiosis in HCV and its hypothetical contribution to the progression of gut–liver axis complications.

*S. salivarius* is significantly enriched during liver cirrhosis and HCC, suggesting that *S. salivarius* plays a pivotal role in the progression of chronic hepatitis into liver cirrhosis and even the development of HCC [144]. *S. salivarius* has down-regulatory impacts on innate immune responses; therefore, their presence may speed up the progression of HCC [151]. Table 1 summarizes the gut microbiota dysbiosis in different stages of HCV infection observed in clinical studies. Moreover, major dysbiosis of gut microbiota during HCV infection and its impact on worsening the disease’s condition and progression to cirrhosis and HCC is summarized in Figure 1.

Therefore, gut dysbiosis is directly linked to the progression of chronic HCV, probably via endotoxemia and hyperammonemia. Thus, gut dysbiosis can be a novel therapeutic target for alleviating the complications of chronic HCV. This can be achieved by either using fecal microbiota transplantation (FMT) or prebiotics and probiotics. Thus, it is not surprising that many reports have recommended the utilization of FMT with treatment regimens of fatty liver disease, PSC, liver cirrhosis, and HCC [154,155,156]. Other studies suggested the incorporation of a broad-spectrum antimicrobial as rifaximin to reduce endotoxemia and harmful metabolites [157,158], as well as to compensate for the reduction in the bile acid levels [97] that all attributed to *Porphyromonadaceae*, *Enterobacteriaceae*, and *Bacteroidaceae* [157]. Thus, there are recommendations for using rifaximin followed by FMT as an augmenting therapy of HCV, stating that this would help get rid of *S. salivarius* accompanied by restoring the healthy microbiota.

Moreover, it is reported that there is no direct effect of HCV treatment using ribavirin (RBV) combined with the immune modulator pegylated interferon (PEG-IFN) on gut dysbiosis [135]. Mechanistically, this regimen elevates bile acid production that is essential for gut homeostasis [97]. Therefore, we can postulate that direct-acting antivirals (DAAs) may correct for the reduced bile acid levels induced by some liver cirrhosis-associated microbiota, including the family of *Enterobacteriaceae*, the genera including *Staphylococcus* and *Enterococcus* [97]. HCV complications treatment can also be enhanced through utilizing *Bifidobacterium* and *lactobacillus acidophilus*, which have probiotic attributes [159]. The beneficial uses of probiotics in treatment HCV-infected patients with cirrhosis are well documented [160]. Moreover, some microbiota can enhance the immune response in HCV patients through activation of CD56+ NK cell counts and CD3+ cells [161]. Enhancement of the cytotoxic activity of NK cells can inhibit HCV replication.

Along with being a novel therapeutic approach, microbiota transition could serve as a biological indicator for the determination of chronic HCV progression. For example, this can be achieved by detecting the significant increase in *Bacteroides* and *Enterobacteriaceae* during mild liver disease or the significant increase in lactobacilli and viridans streptococci are associated with the progression of the disease [112]. Sultan and coauthors have recently identified 5 microbiota OTUs that can differentiate HCV patients from healthy individuals [137].

## 5. Impact of HCV Treatment on Gut Microbiota

The combination of INF-α and RBV was adopted as a standard strategy for controlling chronic HCV in 1999 [162]. In recent years, the short course administration of DAAs has effectively eradicated HCV infection [163]. Consequently, DAAs medications have replaced INF-α and RBV combination as a standard treatment for HCV chronic infections according to the WHO guidelines in 2018 [164]. However, RBV is still recommended to be used with DAAs to treat specific HCV genotypes [164]. The DAAs adopted for HCV treatment are classified according to their viral targets, as mentioned in Table 2 [165]. DAAs are often used in combinations to overcome the development of antiviral resistance [162,166].

HCC is a fatal progressive status of HCV infection. Such a status requires the administration of immune checkpoint inhibitors (ICIs), antibodies (Abs) such as anti-cytotoxic T lymphocyte-associated antigen-4 monoclonal Ab, anti-programmed death 1 Ab, and anti-programmed death ligand 1 Ab [167]. Non-antiviral drugs are also used for the management of HCV extrahepatic manifestations [162]. For example, lactulose is used to improve the cognitive abilities in cirrhotic HCV infected patients [168]. Another example is rituximab, which has been found to be useful for the treatment of HCV-induced cryoglobulinemic vasculitis [169]. Additionally, Korean red ginseng extract improved liver function in patients with chronic HCV infection [170]. Interestingly, the use of probiotics to modulate intestinal microbiota beneficially for human health, e.g., *E. faecalis* strain FK-23, and *L. acidophilus* was significantly useful in cirrhotic HCV-infected patients [69,160].

A few studies investigated the effect of antiviral drugs on gut microbiota of HCV patients [97,135,171]. An early study, conducted by Bajaj et al., investigated the gut microbiota dysbiosis in HCV cirrhosis in patients with/without sustained virological response (SVR) after >1 year of treatment with PEG-IFN and RBV or PEG-IFN + RBV + Telepravir [135]. Gut dysbiosis and proinflammatory responses were reported for all cirrhotic patients regardless of the treatment regimen or SVR. The patients (n = 21) exhibiting SVR in this study belonged to different genotypes; genotype 1 (n = 13), genotype 2 (n = 6), genotype 3 (n = 2).

The study by Ponziani and coauthors demonstrated a significant improvement in the dysbiosis of gut microbiota after successful treatment of HCV-cirrhotic patients via DAAs, which was attributed to the enhancement of liver function resulting from eradication of viral infection [97]. This improvement included an increase in the microbiota diversity to match that of healthy subjects, depletion of HCV-induced increase of potentially pathogenic bacteria, and reduced serum levels of cytokines and chemokines. However, such a favorable impact was independent of the intestinal barrier function, as indicated by the relevant parameters, fecal calprotectin, and plasma levels of circulating zonulin-1 and LPS that remained unchanged after DAAs therapy. The favorable effect of the post-DAAs treatment was indicated by: Firstly, the decrease in the abundance of potentially pathogenic bacteria; *Enterobacteriaceae*, *Enterococcus*, and *Staphylococcus*, due to the increase of bile acid concentration after eradication of infection, secondly, the abundance of *Veillonellaceae*, reported to be increased in case of liver cirrhosis, was also decreased after the treatment of viral infection and thirdly, the increased abundance of *Methanobrevibacter*, where *Methanobrevibacter smithii* is important in bile acids detoxification via the production of bile salt hydrolase [172]. In this study, 12 Caucasian patients from Italy were involved, and the genotypes 1a and 2 were predominant. Moreover, the authors investigated the impact of DAAs treatment on gut microbiota composition one year after the end of DAAs therapy.

Another study, performed by Perez-Matute et al., investigated the effect of DAAs on gut microbiota composition of HCV-infected patients [171]. Only non-cirrhotic patients were included in this work to avoid the direct influence of cirrhosis and portal hypertension on the gut. The HCV-infected patients were evaluated before the treatment, after completing the DAAs regimen, and 12 weeks after ending treatment with SVR. The authors concluded that neither the administration of DAAs nor the 12 weeks in SVR improved the changes caused by HCV at the gut level. In this work, 22 Caucasian patients from Spain were included. Despite the relatively low number of patients involved in this study, all HCV genotypes were included.

The difference in the findings between the study of Perez-Matute et al. and that of Ponziani et al. indicates the relevance of taking the complication status, e.g., cirrhosis, of HCV infection into consideration. Moreover, their conclusions raise fundamental questions about how the treatment of HCV with DAAs might affect gut microbiota composition and gut-liver axis according to the disease’s progression, e.g., acute, chronic infections, or HCC. The impact of DAAs, PEG-IFN, and ribavirin on the gut-microbiota-liver axis and its correlation to the stage of HCV infection, viral genotypes in the previously mentioned studies are summarized in Table 3 [97,135,171].

The administration of lactulose to improve the cognitive abilities and quality of life in cirrhotic patients was associated with alleviating gut microbiota dysbiosis in minimal hepatic encephalopathy [173]. Moreover, lactulose responders differed significantly from non-responders at the phylum level, in Actinobacteria, Bacteroidetes, Firmicutes, and Proteobacteria. On the contrary, Sarangi et al. reported that gut microbiota was not altered after lactulose administration in cirrhotic patients [174]. The authors concluded that the impact lactulose on hepatic encephalopathy may not be related to gut microbiota changes.

The red ginseng extract was reported to improve the composition of gut microbiota via both; enhancing the growth of probiotic bacteria, e.g., *Bifidobacterium* and *Lactobacillus*, and inhibiting the growth of potentially pathogenic bacteria such as *E. coli*, *Staphylococcus aureus*, and *Salmonella* spp. [175,176,177]. It should be mentioned that, all the previous studies regarding red ginseng extract were either performed in vitro or in vivo in animal models. To the best of our knowledge, no studies so far have investigated the effect of red ginseng extract on human gut microbiota.

The effect of immune checkpoint inhibitors (ICIs) on gut microbiota was illustrated recently in HCC patients [178]. The authors found that the diversity of the gut microbiota in HCC who received ICIs were noticeably increased. They concluded that the negative feedback, which is controlled by the interplay between microbial metabolism and host pathways, can be a reason to induce high bacterial diversity. However, it should be mentioned that this study involved HBV-infected patients that suffered from HCC as a serious complication of HBV infection. As far as we can tell, no studies so far have investigated the effect of rituximab on the gut microbiota in HCV-infected patients.

**Table 3:** The impact of different antiviral medications on gut-microbiota-liver axis in previous studies. SVR, sustained viral responses, DAAs, direct-acting antivirals, PEG-IFN, Pegylated interferon, RBV, ribavirin.

Study, (Reference)	Antiviral Regimens	Treatment Duration	Genotype	Country (Race), Infection Stage	Impact of DAA on Gut Microbiota and Gut Liver-Axis	Number of Patients	Number of Controls
Ponziani et al. [97]	Sofosbuvir/ledipasvir, sofosbuvir/RBV, paritaprevir/ritonavir/ombitasvir/dasabuvir or paritaprevir/ritonavir/ombitasvir	1 year after the end of antiviral treatment.	1b and 2 were predominant.	Italy (Caucasian), cirrhotic patients suffering from chronic HCV infection	- Improvement in the gut microbiota diversity, and depletion of potentially pathogenic bacteria. - No effect on the intestinal barrier function.	12	12
Perez-Matute et al. [171]	Ledipasvir + Sofosbuvir, Ombitasvir/Paritaprevir/Ritonavir + RBV, Ombitasvir/Paritaprevir/Ritonavir + Dasabuvir, Sofosbuvir + Daclatasvir, Ombitasvir/Paritaprevir/Ritonavir + Dasabuvir + Ribavirin	After completing the antiviral treatment and 3 months afterthe end of therapy with SVR	1a (5), 1b (9), 2a (1), 2a/2c (1), 3a (3), 4 (3)	Spain (Caucasian), non-cirrhotic patients	- Neither the administration of DAAs nor 3 months in SVR was able to overcome the changes caused by HCV at the gut level.	22	23
Bajaj et al. [179]	PEG-IFN and RBV or PEG-IFN + RBV + Telepravir	>1 year after SVR (A median of 15 months)	1 (13), 2 (6), 3 (2).	The USA, cirrhotic patients, suffering from chronic HCV infection	No significant effect on gut dysbiosis and pro-inflammatory systemic markers after SVR.	21 with SVR 84 without SVR	45

## 6. Gut Microbiota Contribution to Metabolism of HCV Treatment

The individuals vary from each other in terms of their response to medications. A reason for that can be the metabolism of these medications by human gut microbiota [180,181]. Therefore, it is expected that human gut microbiota might play a role in the metabolism of antiviral drugs against HCV infection. Despite the presence of interesting and comprehensive research articles regarding the metabolism of drugs via human gut microbiota [180,181], there is still a shortage of reports concerning antiviral drugs’ metabolism via gut microbiota. Zimmerman and his coauthors have reported that a group of microbiome-generated esterase enzymes (acetyl esterase, lysophospholipase L1, and sialate O-acetylesterase) are involved in antivirals’ metabolism [180]. Still, Further studies are required to identify the full picture of microbiota genes involved in antiviral metabolism, for example, through meta-proteomic and meta-transcriptomics analyses [182]. McCabe et al. demonstrated that deleobuvir was reduced into the metabolite CD 6168, which was mainly generated by gut microbiota metabolism [182]. The authors confirmed this finding via incubation of deleobuvir with human and rat fecal homogenates, in addition to in vivo experiments in a pseudo-germ-free rat model (gut microbiota was mostly eliminated via antimicrobial treatment) in comparison with control rats. Generally, this indicates that the metabolism of antiviral drugs by gut microbiota and the activity of their metabolites should be assessed and taken into consideration in drug development.

## 7. Gut Microbiota Manipulation in HCV Infection

### 7.1. Dietary Intervention-Regimen to Modulate the Gut Microbiota in HCV Patients

Diet plays a pivotal role in shaping the gut microbiota [183]. Factors including environment, family genes, medication use, as well as diet generate a characteristic gut microbiota that differs from one person to another [184]. Fiber-rich diets have a great impact on the enterotype of gut microbiota [185]. Fibers present in diets can only be digested, broken down, and fermented via the gut microbiota enzymes resulting in the release of SCFAs [186]. As a result, pH of the colon decreases, which modulates the composition of microbiota residing in this acidic environment. For example, this acidic environment undermines the growth of some harmful bacteria such as *Clostridium difficile* [187]. In addition, SCFAs have a beneficial impact on health, involving provoking the activity of immune cells and maintaining levels of cholesterol and glucose [186].

Diets that support the elevated levels of SCFAs include indigestible carbohydrates and fibers such as inulin, pectins, gums, resistant starch, and fructo-oligosaccharides [188]. Such fibers are named prebiotics because the beneficial gut microbiota feed on them [188]. Despite many commercial supplements containing these prebiotic fibers, there are many healthy foods that contain such prebiotics, such as the raw version of leeks, garlic, asparagus, onions, artichokes, bananas, dandelion greens, and seaweed [189]. Generally, beans, vegetables, fruits, and whole grains like barley, oats, and wheat are all good sources of prebiotic fibers [188,189]. Diets rich in such prebiotics can be utilized as dietary intervention-regimens to improve the gut microbiota in case of HCV patients [190]. However, caution should be taken during consuming such diets that are rich in prebiotics, because they can lead to bloating and flatulence [191]. Moreover, individuals who suffer from sensitivities including irritable bowel syndrome should take small amounts of these diets firstly to evaluate tolerance [192]. Continuous use of these diets in such patients can enhance the tolerance and decrease the side effects [191,192].

Besides prebiotics, probiotic foods that comprise beneficial live microbiota can be used to modulate the gut microbiota dysbiosis [160]. These involve fermented foods like kefir, miso, kimchi, tempeh, pickled vegetables, kombucha tea, yoghurt with live active cultures, and sauerkraut [193].

Although the impact of prebiotics and probiotics on HCV clinical manifestations is lacking, both prebiotics and probiotic rich diets may modulate the gut microbiota dysbiosis that accompany HCV infection through reducing intestinal permeability, reducing ammonia absorption, and accelerating intestinal transit [194,195,196].

In addition to prebiotics and probiotics, diets that are rich in zinc as well as L-ornithine-L-aspartate (LOLA) are useful as they decrease serum ammonia levels through increasing ammonia detoxification (urea cycle and glutamine synthesis) [197,198]. Vegetable proteins also decrease the serum ammonia levels as they increase ammonia detoxification (urea cycle) and accelerate intestinal transit (high fiber content), as well as reducing circulating mercaptans and indoles [199]. Dairy proteins are also beneficial, as they decrease the serum ammonia levels [199]. Adequate protein intake (1.2–1.5 g/kg per day) decrease the serum ammonia levels by balancing nitrogen metabolism and preventing sarcopenia [197,198,199]. Oral branched-chain amino acids (BCAA) decrease the serum ammonia levels by increasing ammonia detoxification (glutamine synthesis) [197,198,199]. A gluten-casein free diet reduces the absorption of gluten- and casein-derived peptides and decreases the proinflammatory cytokine production [200].

### 7.2. Other Strategies with the Potential of Mitigating Gut Microbiota Dysbiosis in HCV Infection

Bacteriophages, which are also named phages, are the most plentiful organisms in the universe [201]. They are viruses that specifically target bacteria as their host [202]. Bacteriophages can be found in water, sewage, soil, and other places where bacteria tend to be abundant [201]. Bacteriophages infect bacteria, replicate within them, and finally, they lyse the bacterial cells, liberating new phage viruses that tend to infect new bacterial cells and repeat the cycle [202]. Due to their lytic ability and host specificity, bacteriophages have been used as therapeutic approaches for the treatment of bacterial infection a long time ago [201,202]. However, their use as antimicrobials was not on the spot, until recently the problem of multidrug resistance appeared and spread worldwide [201,203].

A prophage is a term related to the inserted integrated genome of the bacteriophage into the host bacterial DNA chromosome [204]. The viral genome occurs within the bacterium genome without leading to bacterial cell lysis until it is specifically activated to produce new phages [205]. Therefore, intervention with prophages at this stage can result in microbiota dysbiosis [205]. Such interferences can be brought about by medications, lifestyle, and diet [184,205]. The management of prophages induction can be utilized as a novel approach to control the human microbiota dysbiosis and is considered as a next-generation phage therapy [206]. One main parameter that enforces the use of bacteriophages as tools for the correction of microbiota dysbiosis and treatment of diseases related to it, is their high bacterial host specificity [201,207]. Therefore, during HCV infections, the gut dysbiosis can be corrected through using specific phages that target the disease associated bacteria [207].

Beside bacteriophages, the immune system of the prokaryotic cells named as CRISPR-Cas allowed researchers to modify and study organisms with exceptional ease and efficiency [208]. CRISPR-Cas systems are defined as the adaptive immune system of bacteria and archaea [209]. The ability of such systems to destroy target nucleic acids, either DNA or RNA, is established via a set of different RNA-guided nucleases [208,209]. Transcription and processing of the CRISPR locus results in production of CRISPR RNAs (crRNA) that guide Cas nucleases [210]. CRISPR locus is defined as the chromosomal site into which invading nucleic acids DNA fragments are incorporated in between repeats, resulting in a memory of past infections [211]. Classification of CRISPR-Cas systems is based on the Cas proteins involved with CRISPR arrays [210,211].

CRISPR-Cas systems are found in nearly 40% of bacteria [209]. Such endogenous CRISPR-Cas systems can be genetically engineered and introduced into target bacteria [208]. Modification of the genomes of microbiota, probiotic bacteria, bacteriophages and yeast can be achieved through using these systems [208]. Getting rid of certain strains can be established through using such systems without affecting the rest of the microbiota [208,209,210,211]. There are three main approaches for microbiota therapies utilizing CRISPR including; additive therapy, subtractive therapy, and modulatory therapy [208]. In additive approaches, designer probiotic strains of bacteria and yeast can be engineered by using CRISPR-Cas systems. In subtractive approaches, elimination of target bacteria can be established utilizing CRISPR-Cas systems, either through the delivery of CRISPR-Cas systems themselves as antimicrobials or the engineering of designer lytic bacteriophages. In modulatory approach, modification of gene expression can be achieved through using dead Cas proteins, and modulation of activity and composition of microbiota can be accomplished through engineered temperate phages.

## 8. Conclusions

**Figure 2:**
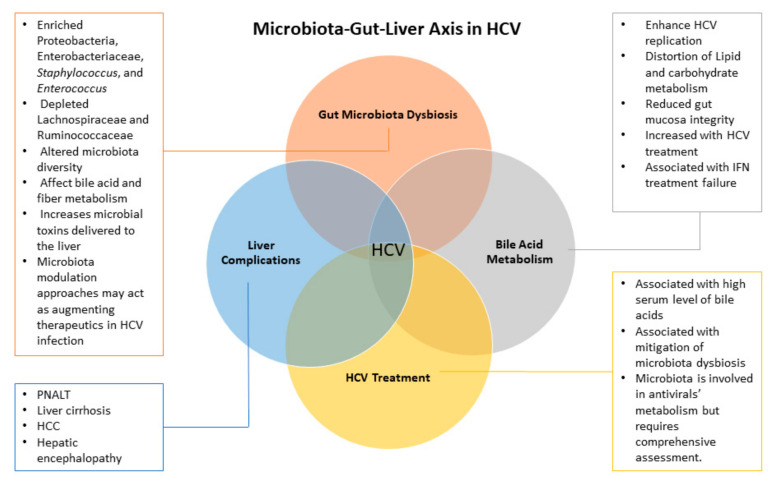
Microbiota–gut–liver axis in HCV infection. HCV is associated with gut microbiota dysbiosis and distortion of bile acid metabolism, which overtime promote liver complications and cross talk with HCV treatment regimens. All these factors affect the enterophepatic axis homeostasis, however, the cause–effect relationship is lacking and requires further comprehensive analysis.

The Gut-liver axis is a bidirectional crosstalk between the gut microbiota and the liver (Figure 2). Gut microbiota is well documented for their beneficial role in human health. Any disturbance in this ensemble is reflected in its functionality and may lead to a health disorder. HCV infection induces physiological changes in both the liver and the intestine, such as bile acid disturbance, which in turn affects the microbiota diversity and structure. Chronic HCV infections are associated with enrichment of potentially harmful bacteria such as *Enterobacteriaceae* and depletion of potentially beneficial microbes such as *Ruminococcaceae* and *Lachnospiraceae*. These changes are related to the progression of the disease and to the studied cohort. Additionally, these changes are thought to promote more disease progression and development of liver complications and intestinal inflammation. Still, the mechanistic understanding of the cause–effect relationship between the microbiota and the disease complications is missing. Additionally, antiviral treatments have their own impact on the gut microbiota, introducing a significant confounding variable that should be controlled. As mentioned above, a recent single study has taken this factor into consideration, and its results showed some contradictions to early literature such as the increase HCV-associated microbial diversity in a treatment naïve cohort. Still, longitudinal analysis of the gut microbiota in HCV patients before starting treatment and over the course of treatment in addition to before and after the virus eradication are needed to get a full picture of the mechanistic association between the liver and the gut microbiota during HCV infection. Additionally, metatranscriptomics along with metabolomics may help reveal the metabolic interaction between the gut microbiota and the anti-HCV treatments.

## Figures and Tables

**Table 1:** Gut microbiota dysbiosis in different stages of hepatitis C virus (HCV) infection.

Stage	Microbiota Dysbiosis	
	Enriched	Depleted
Newly Diagnosed (Treatment-naïve)	*Prevotella*, *Succinivibrio*, *Catenibacterium*, *Megasphaera*, and *Ruminococcaceae* [137].	*Bacteroides*, *Dialister*, *Bilophila*, *Streptococcus*, *Parabacteroides*, *Enterobacteriaceae*, *Erysipelotrichaceae*, *Rikenellaceae* and *Alistipes* [137].
PNALT	*Enterobacteriaceae* [97,123,151]. *Bacterioides* [97,123,151].	
Chronic HCV patients	*Enterobacteriaceae* [97,98,113], Proteobacteria [96,97], Bacterioidetes [112], viridans streptococci [112], *Staphylococcaceae* [96,97], *Enterococcaceae* [96,97].	Firmicutes [112], *Ruminococcaceae* [112] *Lachnospiraceae* [112].
Cirrhotic HCV	*Enterobacteriaceae* [96,97,120], *Veillonellaceae* [120], Proteobacteria [96,97,120], *Bacterioides* [96,122,152], *Staphylococcaceae* [96,97].	*Bacteroidaceae* [120], *Ruminococcaceae* [112], *Lachnospiraceae* [112,120], Firmicutes [112].
HCC	*Streptococcus salivarius* [112,144,151].	*Ruminococcaceae* [112] *Lachnospiraceae* [112].
Hepatic encephalopathy	*Enterobacteriaceae* [122,147,153] *Bacteroides* [147,153].	

**Table 2:** Classification of direct-acting antivirals (DAAs) according to their mechanism of action [165].

NS5A Serine Protease Inhibitor	Protease NS3/4A Inhibitor	NS5B Polymerase Inhibitor (Non-Nucleoside Analogue)	NS5B Polymerase Inhibitor (Nucleotide Analogue)
Daclatasvir	Glecaprevir	Dasabuvir	Sofosbuvir
Elbasvir	Grazoprevir	Deleobuvir	
Ledipasvir	Paritaprevir		
Ombitasvir	Simeprevir		
Pribrentasvir	Voxilaprevir		
Velpatasvir			

## Data Availability

Not applicable.

## Abbreviations

Ab	Antibody
DAAs	Direct acting antiviral drugs
ECM	Extracellular matrix
ER	Endoplasmic reticulum
HAV	Hepatitis A virus
HBV	Hepatitis B virus
HCV	Hepatitis C virus
HEV	Hepatitis E virus
HCC	Hepatocellular carcinoma
HSCs	Hepatic stellate cells
ICIs	Immune checkpoint inhibitors
LPS	Lipopolysaccharides
n	Number
PAMP	Pathogen-associated molecular patterns
PEG-IFN	Pegylated interferon
PNALT	Persistently normal serum alanine aminotransferase stage
PSC	Primary sclerosing cholangitis
RBV	Ribavirin
RdRp	RNA-dependent RNA polymerase
SCFAs	Short-chain fatty acids
SVR	Sustained virological response
UTR	Untranslated region
